# The Relationship between* MC1R* Mutation and Plumage Color Variation in Pigeons

**DOI:** 10.1155/2016/3059756

**Published:** 2016-11-13

**Authors:** Jin-Shan Ran, Xiao-Yan You, Jie Jin, Yu-Guang Zhou, Ye Wang, Dan Lan, Peng Ren, Yi-Ping Liu

**Affiliations:** ^1^Farm Animal Genetic Resources Exploration and Innovation Key Laboratory of Sichuan Province, Sichuan Agricultural University, Chengdu Campus, Chengdu 611130, China; ^2^Chongqing Academy of Animal Science, Rongchang, Chongqing 402460, China

## Abstract

The polymorphisms of* MC1R* gene play a crucial role in coat color variation in mammals; however, the relationship is still unclear in pigeons. In this study, we sequenced 741 bp fragment of the* MC1R* for 39 individuals with five plumage color patterns (gray plumage, *n* = 12; black plumage, *n* = 9; white plumage, *n* = 3; spotted plumage, *n* = 12; red plumage, *n* = 3). A total of three single nucleotide polymorphisms (SNPs) were detected, including G199A, G225A, and A466G, which subsequently determined four haplotypes (H1–H4). Among them, H1 is the predominant haplotype. Association analysis revealed that H1 and H3 were significantly associated with the black plumage trait (*P* < 0.05), while the H4 was significantly associated with gray plumage trait (*P* < 0.05). Furthermore, only diplotype H1H1 was significantly associated with black and gray traits of pigeons. Collectively, our study suggested an association between genetic variation of* MC1R* and plumage color in pigeon.

## 1. Introduction

The coat and plumage color of mammals and birds are mainly related to the pigment distribution or proportion of eumelanin and pheomelanin [1]. The relative ratio of eumelanin and pheomelanin is regulated by melanocortin receptor-1 (MC1R) and its antagonist agouti protein [2]. MC1R encodes a seven-transmembrane domain G-protein-coupled receptor, expressed primarily in melanocytes of developing feathers and hair [3]. The process of eumelanin synthesis is triggered by the binding of *α*-melanocyte stimulating hormone (*α*-MSH) to MC1R [4]. Then, it will lead to an increase of intracellular cAMP which activates tyrosinase and catalyzes the first step of melanogenesis [5].* MC1R* mutations causing a constitutively active receptor are dominant and associated with black color, while loss-of-function mutations are recessive and associated with a red/yellow phenotype [6].

In previous studies, it has been confirmed that mutations of the* MC1R* gene were associated with melanin trait variation or skin cancer and other diseases in a number of mammalian species, such as human, mouse, cattle, horse, fox, pig, sheep, and dog [7–16]. In birds, studies on molecular mechanism of melanin deposition are relatively rare at present. According to the same pigment variation resulted from the same* MC1R* mutationsin in the chicken and mouse, it speculated that the function of* MC1R* in regulating mechanism is likely to be consistent in chicken and mammals [17]. The same studies are also found in duck, goose, swan, and bananaquit [3, 18–20]. More and more avian* MC1R* gene has been cloned; however, the potential association between* MC1R* gene and plumage color phenotypes in pigeons has not been validated. The aim of the current study is to detect SNPs in* MC1R* gene and explore their phenotypes association with plumage color in pigeons.

## 2. Material and Methods

### 2.1. Samples

**Figure 1:**
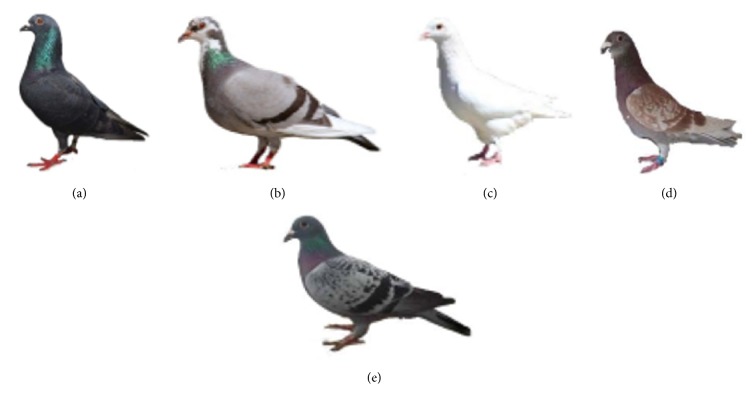
The plumage color of (a) black plumage, (b) gray feather, (c) white plumage, (d) red feather, and (e) spotted plumage.

Blood samples were collected from 39 pigeons (Aplopelia Bonaparte) with five plumage color phenotypes, including 9 black plumage pigeons (pure black feather), 12 gray plumage pigeons (completely gray plumage), 3 white plumage pigeons (pure white plumage), 3 red plumage pigeons (completely gray feather), and 12 spotted feather pigeons (as the raindrops distribution in the body) (Figure 1). The pigeons came from the poultry market of Ya'an. The protocol was approved by the Committee on the Care and Use of Laboratory Animals of the State-Level Animal Experimental Teaching Demonstration Center of Sichuan Agricultural University. All samples were immediately refrigerated with drikold before transferred and stored at −20°C.

### 2.2. DNA Extraction and PCR Amplification

DNA was extracted from blood samples using QIAamp DNA Blood Mini Kit according to the instructions. DNA was eluted in a final volume of 200 *μ*L using AE buffer and then stored at −20°C. The primer pair of* MC1R* gene (F: 5′-GCC AGC GAG GGC AAC CAG AGC-3′; R: 5′-AAG GGG TTG GTG GGG CAG GTG ACG A-3′) were designed by Primer BLAST on NCBI according to the rock pigeon reference sequence (GenBank accession NW_004973333.1).

The PCR reaction (25 *μ*L) contains 12.5 uL 2×Taq MasterMix, 1 uL forward primer (10 uM), 1 uL reverse primer (10 uM), 2 uL DNA, and 8.5 uL ddH2O. PCR cycles included 95°C for 5 min; 35 cycles included 95°C for 30 s, 58.5°C for 30 s, and 72°C for 90 s; and a final extension included 12°C for 10 min, ending with an incubation at 4°C.

PCR products were checked on 1% agarose gel and sequenced by TSINGKE Biological Technology Corporation.

### 2.3. Sequence Analysis

All sequences were spliced and aligned by DNAstar package (DNASTAR Inc., Madison, WI, USA). We predicted secondary structure of MC1R protein by PSIPRED protein structure prediction server (http://bioinf.cs.ucl.ac.uk/psipred/) [21]. We used MEGA 5.1 to export sequence variations. Haplotypes of the* MC1R* gene were deduced by using the PHASE 2.0 program. The potential association between the* MC1R* alleles and plumage colors was evaluated by chi-square test for independence which was performed on SAS V8.1 (SAS Institute Inc., Cary, NC, USA).

## 3. Results

### 3.1. Sequence Polymorphism in the* MC1R* Gene

We obtained 741 bp fragments of* MC1R* gene in this study. Three SNPs (G199A, G225A, and A466G) were detected from* MC1R* gene: one of them was synonymous and two of them were leading to amino acid substitution (Asp67Asn and Thr156Ala). All of these SNPs were newly reported.

### 3.2. MC1R Protein Secondary Structure Prediction

**Figure 2:**
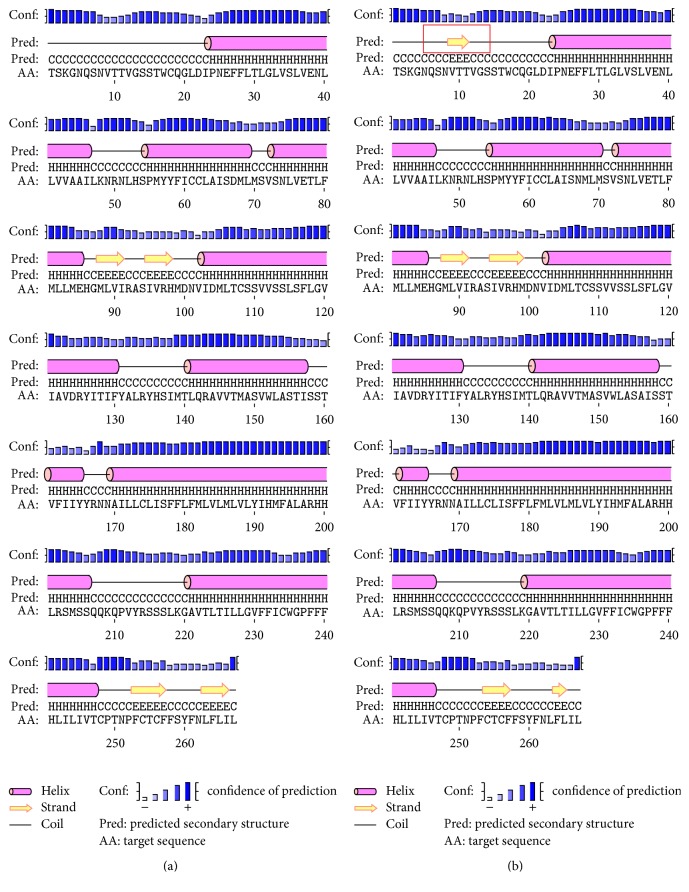
Secondary structure prediction of* MC1R* protein ((a) for nonmutated sequence; (b) for mutated sequence).

According to the sequence of amplification, we predicted nonmutated and nonsynonymous mutated of MC1R protein secondary structure, respectively (Figure 2).

### 3.3. Allele and Genotype Frequency of the Mutated Loci

The results of the allele and genotype frequency of the 3 SNPs in population were shown in Table 1. The chi-squared test was used to compare the allele frequencies in the* MC1R* gene between the different plumage color groups. Three mutations of different genotype distribution in pigeons of the five types of plumage color difference reached significant level (*P* < 0.05). In the group, SNP1 (G199A) and SNP2 (G225A) showed a homozygous genotype GG for advantage genotype, while SNP3 (A466G) showed AA genotype for advantage. To associate different plumage color pattern with genotypes composed of SNP1 and SNP2 loci, the results show that GG genotype was the advantage genotype in black, gray, and spotted pigeons, and AG genotype in gray and spotted feather was predominated. For SNP3 loci, AA genotype was the advantage genotype in black, gray, and spotted pigeons; heterozygous genotype AG occupied absolute advantage in black and spotted plumage.

### 3.4. Haplotype Analysis

Four haplotypes (H1–H4) were obtained based on the three SNPs (G199A, G225A, and A466G) of the* MC1R* gene (Table 2), and the corresponding diplotypes were displayed in Table 3. The results showed that haplotype H2 was only found in the spotted feather and haplotype H1 was found in all kinds of feather colors with a high frequency. Particularly, haplotype H4 occurred in gray feather and spotted feather with the twice frequency. Association analysis demonstrated that haplotypes H1 and H3 were significantly associated with the black plumage trait of pigeons, whereas the H1, H3, and H4 were significantly associated with gray and spotted plumage traits of pigeons (*P* < 0.05). Furthermore, the diplotypes H1H4 and H3H3 were only distributed in gray feather, and the diplotype H2H2 was only distributed in spotted plumage. Association analysis showed that diplotype H1H1 was significantly associated with black and gray traits of pigeons (*P* < 0.05).

## 4. Discussion

The plumage color of poultry has been widely used as a morphological marker for genetic selection and considered as an important economic trait that caters to consumer preference also. The* MC1R* gene polymorphisms have impacts on the plumage colors and skin traits in domestic animals [22, 23]; different alleles of the* MC1R* gene were associated with red hair color, fair skin, and skin cancer risk in human [24]. In our previous study, we found that there was significant association between the MC1R genetic variation and plumage colors of chicken and geese [25, 26].

In the past 10 years, lots of* MC1R* gene mutation and the feather color difference relations research could form the perspective of the molecular to expound melanin synthesis regulative process, and for birds and mammals the molecular mechanism on the formation of melanin provides a solid theoretical basis. In this study, we identified two missense and one synonymous mutations in 39 samples. The SNP1 (G199A) and SNP3 (A466G) lead to a change in amino acids (Asp67Asn and Thr156Ala). In genetics, a missense mutation, a type of nonsynonymous substitution, could result in truncation of the resulting protein and protein nonfunctional. Through the protein secondary structure prediction, we can clearly see that one more strand was found in nonsynonymous mutations MC1R protein secondary structure which may affect the function or efficiency of the protein. When the amino acid loci 156 of MC1R protein mutated into alanine, pigeon plumage color occurs as albino (grey, grizzle, and white); this result is 156 similar to Guernsey's research [27]. Derelle et al. reported 10 nonsynonymous mutations, while they found no association with plumage changes in feral pigeons; this result is not consistent with our study [28]. We speculated that the difference between sample size and variety led to this outcome. In addition, although the one SNP did not cause amino acid change, association analysis showed that they were significantly associated with pigeon plumage colors (*P* < 0.05). Previous studies have shown that different synonymous degenerate codon on these loci would affect protein translation efficiency and structural conformation, which finally lead to phenotypic changes [29–31]. We speculated that this one synonymous variant may impact the function of the MC1R transmembrane domain conformation and activity. A silent (synonymous) mutation in a complex membrane transport protein alters the substrate specificity. Anthony's study hypothesized that when frequent codons are changed to rare codons in a cluster of infrequently used codons, the timing of cotranslational folding is affected and may result in altered function [32]. For the sample size which is relatively limited, further more studies should be carried forward on the relationship between loci and plumage color traits in pigeon.

For the purpose of investigating the possible function of the mutations, we analyzed the association between* MC1R* genotypes with plumage color trait. Haplotype analysis provided a practical solution to resolve these problems. Haplotypes were constructed with the three SNPs and were used to analyze the association of haplotypes with plumage color traits. We found that haplotype H1 was distributed in all kinds of feather colors with a high frequency and was significantly associated with the black plumage trait of pigeon. We also found that diplotypes were significantly associated with plumage colors. The diplotype H2H2 was only distributed in spotted plumage, while the H1H1 was significantly associated with black and gray traits of pigeon. These results demonstrated that haplotypes and diplotypes of the* MC1R* gene bear the characteristic of regional distribution and were associated with plumage color in pigeon, although the plumage color control is a very complex trait.

## 5. Conclusion

On the basis of our results in this study, we speculated that there are significant associations between plumage colors and genetic variants of the* MC1R* gene in pigeon. However, as a complex trait, plumage color is determined by a complex pathway system and multiple interactive patterns; further studies would be helpful to confirm this conclusion.

## Figures and Tables

**Table 1:** The genotype distribution of *MC1R* gene.

SNPs	Genotype	Phenotype class					Total	Frequency/%	*χ* ^2^ value	PIC^1^
		Black	Gray	White	Spotted	Red				
SNP1 (G199A)	GG	9 (0.23)	10 (0.26)	3 (0.08)	11 (0.28)	3 (0.08)	36 (0.92)	92.31	*χ* ^2^ = 57.96 *P* < 0.05	0.1215
	AG	0 (0.00)	2 (0.05)	0 (0.00)	1 (0.03)	0 (0.00)	3 (0.08)	7.69		
	AA	0 (0.00)	0 (0.00)	0 (0.00)	0 (0.00)	0 (0.00)	0 (0.00)	0.00		
	Total	9	12	3	12	3	39	100		
	G	18	22	6	23	6	75	96.15		
	A	0	2	0	1	0	3	3.85		

SNP2 (G225A)	GG	7 (0.18)	10 (0.26)	3 (0.08)	9 (0.23)	3 (0.08)	32 (0.82)	82.05	*χ* ^2^ = 61.32 *P* < 0.05	0.2136
	AG	2 (0.05)	1 (0.03)	0 (0.00)	3 (0.08)	0 (0.00)	6 (0.15)	15.38		
	AA	0 (0.00)	1 (0.03)	0 (0.00)	0 (0.00)	0 (0.00)	1 (0.03)	2.56		
	Total	9	12	3	12	3	39	100		
	G	16	21	6	21	6	70	90.91		
	A	0	3	0	3	0	8	9.09		

SNP3 (A466G)	GG	0 (0.00)	1 (0.03)	0 (0.00)	1 (0.03)	0 (0.00)	2 (0.05)	5.13	*χ* ^2^ = 72.51 *P* < 0.05	0.2652
	AG	2 (0.05)	1 (0.03)	0 (0.00)	3 (0.08)	0 (0.00)	6 (0.15)	15.38		
	AA	7 (0.18)	10 (0.26)	3 (0.08)	8 (0.21)	3 (0.08)	31 (0.79)	79.49		
	Total	9	12	3	12	3	39	100		
	G	2	3	0	5	0	10	12.82		
	A	16	21	6	19	6	68	87.18		

^1^PIC = polymorphism information content. PIC > 0.5 indicating a high level of polymorphism, 0.25 < PIC < 0.5 indicating a medium level of polymorphism, and PIC < 0.25 indicating a low level of polymorphism.

**Table 2:** Distribution of the *MC1R* gene haplotypes.

Haplotype	Number	Frequency	Black	Gray	White	Spotted	Red
H1 (GGA)	28	0.72	0.21 (8)	0.23 (9)	0.08 (3)	0.18 (7)	0.02 (1)
H2 (GGG)	1	0.03	0	0	0	0.03 (1)	0
H3 (GAG)	7	0.18	0.05 (2)	0.05 (2)	0	0.08 (3)	0
H4 (AGA)	3	0.07	0	0.05 (2)	0	0.02 (1)	0

Total	39	1.00					

**Table 3:** Distribution of the *MC1R* gene diplotypes.

Diplotype	Number	Frequency	Black	Gray	White	Spotted	Red
H1H1	28	0.72	8	9	3	7	1
H1H3	6	0.14	4	0	0	2	0
H1H4	1	0.03	0	1	0	0	0
H2H2	1	0.03	0	0	0	1	0
H3H3	1	0.03	0	1	0	0	0
H3H4	2	0.05	0	1	0	1	0

Total	39	1.00	12	12	3	11	1
